# Crystal structure of bis­(diiso­propyl­amino)­fluoro­borane

**DOI:** 10.1107/S2056989025003160

**Published:** 2025-04-17

**Authors:** Tabea Lenz, Marian Hebenbrock

**Affiliations:** a University of Münster, Institute of Inorganic and Analytical Chemistry, Corrensstrasse 30, 48149 Münster, Germany; Texas A & M University, USA

**Keywords:** crystal structure, fluoro­borane,

## Abstract

The predominantly planar structure of a fluoro-substituted bis­(di­alkyl­amino)­borane, C_12_H_28_BFN_2_, was obtained from the reaction of boron trifluoride diethyl etherate with lithium diiso­propyl­amide and its structure is presented here. While the B—F bond length is in the typical range of single B—F bonds, the B—N bond length indicates a partial double-bond character. The sterically demanding isopropyl groups on both amides increase the N—B—N angle and enable inter­molecular van der Waals inter­actions.

## Abstract 2

Bis(diiso­propyl­amino)­fluoro­borane was previously identified as a product of reactions of (diiso­propyl­amino)­difluoro­boranes in the presence of Na/K alloy, but its structure was never elucidated. The structure reported here will contribute to the understanding of the basic binding situation and properties, and will provide a more com­plete picture when com­pared with analogous structures.

## 1. Chemical context

Bis(di­alkyl­amino)­boranes and their derivatives have emerged as prominent starting materials in the field of boron chemistry due to their ease of functionalization and enhanced stability, attributable to the double-bond character of the B—N bond. The fact that these substituents can also be used to realize unusual coordination patterns on the B atom was first demonstrated in 1982 by Nöth and Parry through the formation of the two-coordinate borinium cation (Nöth *et al.*, 1982 ▸; Higashi *et al.*, 1982 ▸). Nöth generated the borinium cations from bis­(di­alkyl­amino)­bromo­boranes by reaction with Lewis acids, while Parry used analogous chloro­boranes. This pioneering work was further expanded, exploring the use of a variety of amines, Lewis acids and boranes (Nöth *et al.*, 1984 ▸, 1986 ▸; Kölle *et al.*, 1986 ▸). More recently, Major *et al.* (2019 ▸) have demonstrated an alternative approach based on analogous fluorinated com­pounds, wherein a silylium cation functions as a fluoride abstractor. They also showed that these borinium cations are versatile reagents in hyroboration reactions. Here we report the structure of the starting material, bis­(diiso­propyl­amino)­fluoro­borane, **1**, which was also previously identi­fied as a product of reactions of (di­iso­propyl­amino)­difluoro­boranes in the presence of Na/K alloy (Maringgele *et al.*, 1992 ▸, 1993 ▸), but its structure was never elucidated. The structural investigation of such a simple com­pound will contribute to the understanding of the basic binding situation and properties, and will provide a more com­plete picture when com­pared with analogous structures.
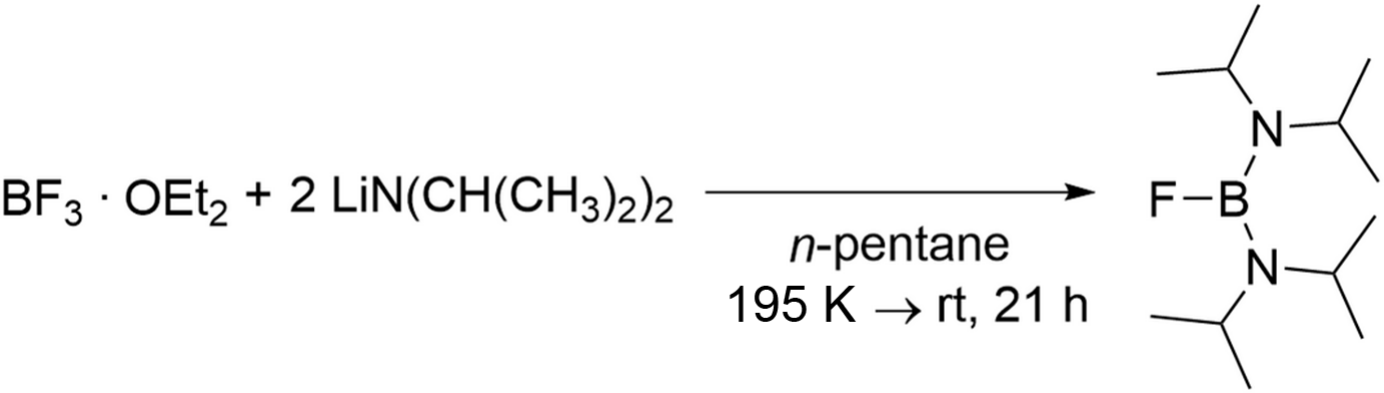


## 2. Structural commentary

**Figure 1:**
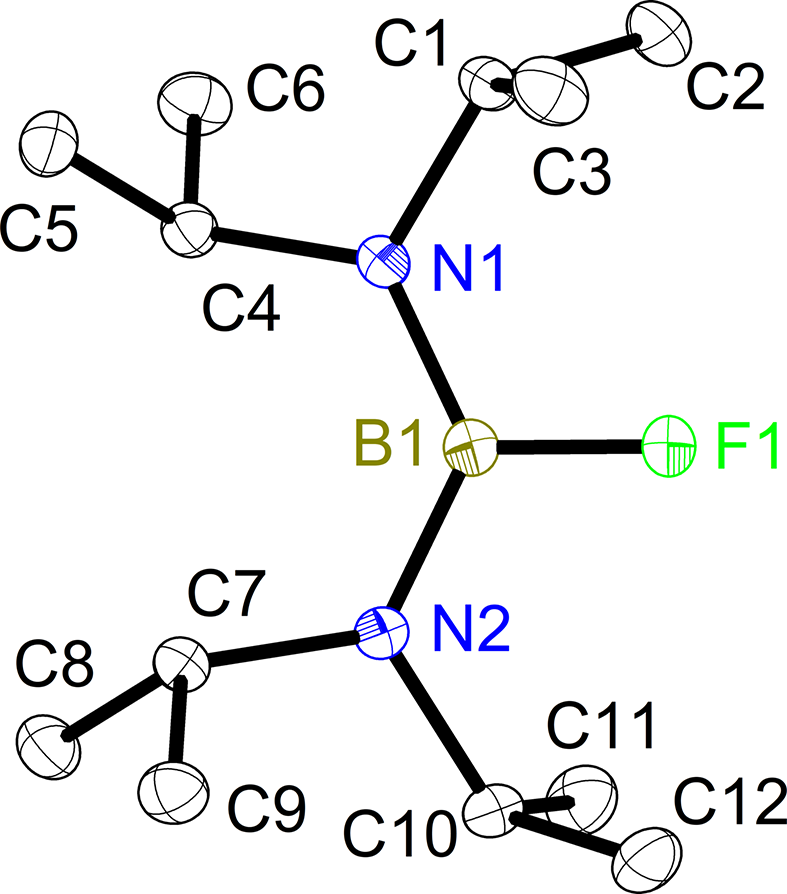
The asymmetric unit of the solid-state structure of com­pound **1**, with the atom-labelling scheme. Displacement ellipsoids are shown at the 50% probability level and H atoms have been omitted for clarity.

Compound **1** crystallizes in the triclinic space group *P*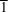 and has one mol­ecule in the asymmetric unit (Fig. 1 ▸). The bond length between boron and fluorine [1.3650 (9) Å] falls within the typical range of bond lengths observed for B—F bonds. The B—N bond lengths [1.4227 (10) and 1.4206 (10) Å] not only correspond to typical boron alkyl­amides, but also show a bond length resulting from the partial formation of a B—N double bond. The N—B—N angle [128.91 (7)°] exhibits a slight increase com­pared to analogous com­pounds. This is attributed to the sterically demanding substituents present on both amines, along with the fluorine substituent, which exhibits a smaller steric bulkiness, thereby enabling the widening of the bond angle. The overall structure of the com­pound is predominantly planar, which is consistent with the expected geometry of a trigonal-substituted B atom with partial double bonds to the substituents.

## 3. Supra­molecular features

**Figure 2:**
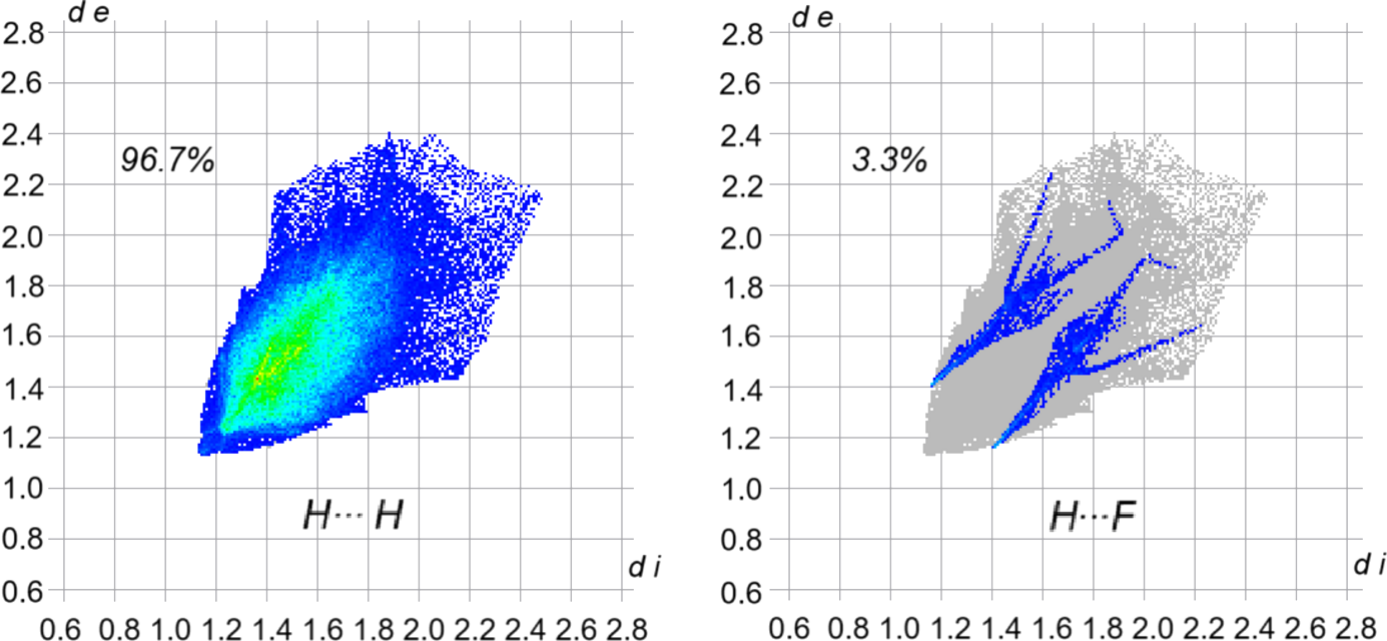
Decom­posed two-dimensional fingerprint plots of the inter­actions of com­pound **1** devided in reciprocal H⋯H (left) and H⋯F (right) contacts along with their contributions (*CrystalExplorer*; Spackman *et al.*, 2021 ▸).

**Figure 3:**
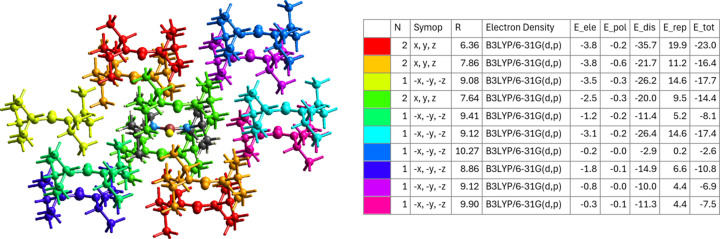
Inter­action energies (in kJ mol^−1^) for different contacts within the structure of **1**, divided into electrostatic, polarization, dispersion and repulsive contributions, together with the total inter­action energy. In the partial packing diagram (left), the inter­acting mol­ecules are colour-coded to correlate with the tabulated energies (*CrystalExplorer*; Spackman *et al.*, 2021 ▸).

Compound **1** does not show any significant inter­molecular inter­actions. The formation of four-membered rings, as found for example in analogous amino­boron difluorides (Hazell, 1966 ▸; Edwards *et al.*, 1970 ▸; Jones, 1984 ▸), is not possible in the case of com­pound **1** due to the steric demand of the isopropyl side groups. The isopropyl groups themselves are capable of significant van der Waals inter­actions. These inter­actions were evaluated using the *CrystalExplorer* program (Spackman *et al.*, 2021 ▸). It was found that the inter­actions occur almost exclusively through H⋯H contacts, as is typical for van der Waals inter­actions (Fig. 2 ▸). A small fraction is also due to H⋯F inter­actions. Inter­actions *via* the N atoms or the B atom do not take place. A decom­position of the inter­action energy between the mol­ecules reveals that the inter­action is predominantly mediated by dispersion forces, with electrostatic or polarization com­ponents being negligible (Fig. 3 ▸).

## 4. Database survey

A database search [Cambridge Structural Database (CSD), Version 5.45, update June 2024; Groom *et al.*, 2016 ▸] for analogous com­pounds reveals only two di­amino­fluoro­boranes with acyclic amines (CSD refcodes YUBMUE and YUBNEP; Ott *et al.*, 2009 ▸). The major difference between these com­pounds and com­pound **1** is that only one of the amines has two isopropyl substituents, while the other amine is substituted with an aromatic substituent and a proton. The B—F bond lengths in both structures range from 1.363 to 1.368 Å, the B—N bonds to the isopropyl-substituted amines range from 1.395 to 1.410 Å and the N—B—N bond angles range from 126.90 to 128.21°. Furthermore, an expanded search, encom­passing diiso­propyl­amine-substituted trigonal–planar boranes in general, yields 351 entries, with the B—N bond lengths ranging from 1.316 to 1.501 Å, contingent on the specific substituents on the B atom. Subsequent to quaternization of the N atom by protonation, a substantial elongation of the B—N bond is observed (1.571 Å in YUBNOZ; Ott *et al.*, 2009 ▸), attributable to the elimination of the partial double-bond character.

## 5. Synthesis and crystallization

### 5.1. General considerations

All reagents were purchased from commercial suppliers and used without further purification. Pentane was dried using lithium aluminium hydride and distilled before use. Reactions of the air-sensitive com­pounds were carried out under an inert argon atmosphere using the Schlenk line technique. NMR spectra were recorded on a Bruker Avance (Neo) 500 instruments. NMR spectra were referenced to residual solvent peaks (C_6_D_6_). Mass spectra were recorded on a Bruker Impact II instrument. The single-crystal X-ray diffraction (SC-XRD) data were collected on a Bruker Venture with a Photon III CMOS detector with Mo *K*α radiation (λ = 0.71073 Å). The experimental procedure was adapted from Major *et al.* (2019 ▸).

### 5.2. Experimental procedure

Boron trifluoride diethyl etherate (1.63 ml, 13.0 mmol) was dissolved in pentane (50 ml) and the solution cooled to 195 K. A solution of lithium diiso­propyl­amide in THF (13.0 ml, 26.0 mmol, 2 *M*) was then added dropwise. The resulting solution was stirred for 7 h at 195 K and then for 14 h at room tem­per­a­ture. A precipitated orange solid was separated by filtration and the resulting solution was concentrated *in vacuo* at 273 K. The residue was redissolved in *n*-pentane (7 ml) and stored at 247 K for 2 d. The suspension was then cooled to 195 K and the precipitated yellowish solid was separated by filtration. The solvent was removed *in vacuo* and the uptake in pentane and subsequent filtration were repeated as described above. Compound **1** was obtained as a yellowish crystalline solid (yield: 1.13 g, 4.91 mmol). Colourless crystals suitable for X-ray crystallography were obtained from the solid by sublimation (yield 38%). ^1^H NMR (500 MHz, C_6_D_6_): δ (ppm) 3.20 [hept, ^3^*J*_HH_ = 6.7 Hz, 4H, NC*H*(CH_3_)_2_], 1.18 [*d*, ^3^*J*_HH_ = 6.9 Hz, 24H, NCH(C*H*_3_)_2_]. ^13^C{^1^H} NMR (101 MHz, C_6_D_6_): δ (ppm) 45.3 [N*C*H(CH_3_)_2_], 23.9 [*d*, ^4^*J*_CF_ = 2.5 Hz, NCH(*C*H_3_)_2_]. ^11^B{^1^H} NMR (160 MHz, C_6_D_6_): δ (ppm) 25.0 (*s*). ^19^F{^1^H} NMR (471 MHz, C_6_D_6_): δ (ppm) −108.9 (*s*).

## 6. Refinement

Crystal data, data collection and structure refinement details are summarized in Table 1 ▸. H atoms were placed at ideal calculated positions and refined using a riding model.

## Supplementary Material

Crystal structure: contains datablock(s) I. DOI: 10.1107/S2056989025003160/jy2057sup1.cif

Structure factors: contains datablock(s) I. DOI: 10.1107/S2056989025003160/jy2057Isup2.hkl

Supporting information file. DOI: 10.1107/S2056989025003160/jy2057Isup3.cml

CCDC reference: 2441918

Additional supporting information:  crystallographic information; 3D view; checkCIF report

## Figures and Tables

**Table 1:** Experimental details

Crystal data	
Chemical formula	C_12_H_28_BFN_2_
*M*_r_	230.17
Crystal system, space group	Triclinic, *P* 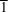
Temperature (K)	100
*a*, *b*, *c* (Å)	6.3603 (2), 7.6440 (3), 16.4098 (6)
α, β, γ (°)	84.334 (1), 84.820 (1), 67.518 (1)
*V* (Å^3^)	732.38 (5)
*Z*	2
Radiation type	Mo *K*α
μ (mm^−1^)	0.07
Crystal size (mm)	0.50 × 0.50 × 0.10

Data collection	
Diffractometer	Bruker D8 VENTURE with a PHOTON III CMOS detector
Absorption correction	Empirical (using intensity measurements) (*SADABS*; Bruker, 2021 ▸)
*T*_min_, *T*_max_	0.514, 0.746
No. of measured, independent and observed [*I* > 2σ(*I*)] reflections	44165, 4103, 3776
*R*_int_	0.043
(sin θ/λ)_max_ (Å^−1^)	0.695

Refinement	
*R*[*F*^2^ > 2σ(*F*^2^)], *wR*(*F*^2^), *S*	0.042, 0.110, 1.08
No. of reflections	4103
No. of parameters	153
H-atom treatment	H-atom parameters constrained
Δρ_max_, Δρ_min_ (e Å^−3^)	0.51, −0.27

Computer programs: *SAINT-Plus* (Bruker, 2021 ▸), SHELXT2018 (Sheldrick, 2015*a* ▸), *SHELXL2019* (Lübben *et al.*, 2019 ▸; Sheldrick, 2015*b* ▸), *SHELXTL* (Sheldrick, 2008 ▸), *APEX4* (Bruker, 2021 ▸) and *publCIF* (Westrip, 2010 ▸).

## supplementary crystallographic information

### Bis(diisopropylamino)fluoroborane Crystal data

**Table:**

C_12_H_28_BFN_2_	*Z* = 2
*M**_r_* = 230.17	*F*(000) = 256
Triclinic, *P*1	*D*_x_ = 1.044 Mg m^−^^3^
*a* = 6.3603 (2) Å	Mo *K*α radiation, λ = 0.71073 Å
*b* = 7.6440 (3) Å	Cell parameters from 9941 reflections
*c* = 16.4098 (6) Å	θ = 2.5–29.6°
α = 84.334 (1)°	µ = 0.07 mm^−^^1^
β = 84.820 (1)°	*T* = 100 K
γ = 67.518 (1)°	Plate, colourless
*V* = 732.38 (5) Å^3^	0.50 × 0.50 × 0.10 mm

### Bis(diisopropylamino)fluoroborane Data collection

**Table:**

Bruker D8 VENTURE with a PHOTON III CMOS detector diffractometer	3776 reflections with *I* > 2σ(*I*)
Radiation source: microsource	*R*_int_ = 0.043
f\ and w\ scans	θ_max_ = 29.6°, θ_min_ = 2.5°
Absorption correction: empirical (using intensity measurements) (SADABS; Bruker, 2021)	*h* = −8→8
*T*_min_ = 0.514, *T*_max_ = 0.746	*k* = −10→10
44165 measured reflections	*l* = −22→22
4103 independent reflections	

### Bis(diisopropylamino)fluoroborane Refinement

**Table:**

Refinement on *F*^2^	Primary atom site location: intrinsic phasing
Least-squares matrix: full	Hydrogen site location: inferred from neighbouring sites
*R*[*F*^2^ > 2σ(*F*^2^)] = 0.042	H-atom parameters constrained
*wR*(*F*^2^) = 0.110	*w* = 1/[σ^2^(*F*_o_^2^) + (0.0628*P*)^2^ + 0.1203*P*] where *P* = (*F*_o_^2^ + 2*F*_c_^2^)/3
*S* = 1.08	(Δ/σ)_max_ = 0.001
4103 reflections	Δρ_max_ = 0.51 e Å^−^^3^
153 parameters	Δρ_min_ = −0.27 e Å^−^^3^
0 restraints	

### Bis(diisopropylamino)fluoroborane Special details

**Table:**

Geometry. All esds (except the esd in the dihedral angle between two l.s. planes) are estimated using the full covariance matrix. The cell esds are taken into account individually in the estimation of esds in distances, angles and torsion angles; correlations between esds in cell parameters are only used when they are defined by crystal symmetry. An approximate (isotropic) treatment of cell esds is used for estimating esds involving l.s. planes.

### Bis(diisopropylamino)fluoroborane Fractional atomic coordinates and isotropic or equivalent isotropic displacement parameters (Å^2^)

**Table:**

	*x*	*y*	*z*	*U*_iso_*/*U*_eq_	
F1	0.93904 (9)	0.04267 (7)	0.71623 (3)	0.02497 (13)	
N1	0.88667 (11)	0.25106 (9)	0.81925 (4)	0.01548 (13)	
N2	0.72717 (11)	0.36432 (9)	0.67705 (4)	0.01613 (14)	
C1	0.94682 (13)	0.09107 (10)	0.88255 (4)	0.01678 (15)	
H1	0.948918	0.145510	0.935300	0.020*	
C2	1.18385 (14)	−0.06119 (12)	0.86768 (5)	0.02304 (17)	
H2A	1.224662	−0.148577	0.916731	0.035*	
H2B	1.295300	−0.000875	0.855770	0.035*	
H2C	1.183618	−0.131787	0.821000	0.035*	
C3	0.76418 (15)	0.00544 (12)	0.89528 (5)	0.02429 (17)	
H3A	0.803276	−0.093603	0.940334	0.036*	
H3B	0.755731	−0.049951	0.844889	0.036*	
H3C	0.616325	0.104925	0.908771	0.036*	
C4	0.88051 (12)	0.43069 (10)	0.84597 (4)	0.01606 (15)	
H4	0.845794	0.524505	0.797241	0.019*	
C5	0.69137 (14)	0.51110 (11)	0.91219 (5)	0.02059 (16)	
H5A	0.679720	0.638557	0.922731	0.031*	
H5B	0.726963	0.428370	0.962753	0.031*	
H5C	0.546150	0.517926	0.893549	0.031*	
C6	1.11067 (14)	0.41371 (12)	0.87467 (5)	0.02167 (16)	
H6A	1.103194	0.539362	0.886261	0.033*	
H6B	1.229965	0.362372	0.831561	0.033*	
H6C	1.146032	0.328549	0.924554	0.033*	
C7	0.54639 (12)	0.54721 (10)	0.69565 (4)	0.01650 (15)	
H7	0.528695	0.550183	0.756731	0.020*	
C8	0.60564 (14)	0.71800 (11)	0.66229 (5)	0.02176 (16)	
H8A	0.486698	0.834686	0.681208	0.033*	
H8B	0.615843	0.724314	0.602201	0.033*	
H8C	0.752295	0.704329	0.682121	0.033*	
C9	0.31532 (13)	0.56770 (12)	0.66626 (5)	0.02146 (16)	
H9A	0.197235	0.685037	0.684966	0.032*	
H9B	0.277831	0.458913	0.688826	0.032*	
H9C	0.323321	0.572230	0.606207	0.032*	
C10	0.76673 (13)	0.32376 (11)	0.58934 (4)	0.01805 (15)	
H10	0.680044	0.444801	0.557470	0.022*	
C11	1.01744 (15)	0.26992 (13)	0.56098 (5)	0.02464 (17)	
H11A	1.036191	0.253792	0.501909	0.037*	
H11B	1.108985	0.150774	0.590426	0.037*	
H11C	1.068066	0.370637	0.572384	0.037*	
C12	0.67586 (15)	0.17610 (13)	0.56824 (5)	0.02438 (17)	
H12A	0.687330	0.168847	0.508612	0.037*	
H12B	0.516015	0.212989	0.588332	0.037*	
H12C	0.766010	0.051867	0.594240	0.037*	
B1	0.84742 (14)	0.22573 (12)	0.73804 (5)	0.01686 (16)	

### Bis(diisopropylamino)fluoroborane Atomic displacement parameters (Å^2^)

**Table:**

	*U*^11^	*U*^22^	*U*^33^	*U*^12^	*U*^13^	*U*^23^
F1	0.0343 (3)	0.0151 (2)	0.0207 (2)	−0.0022 (2)	−0.00725 (19)	−0.00367 (17)
N1	0.0176 (3)	0.0128 (3)	0.0152 (3)	−0.0044 (2)	−0.0035 (2)	0.0002 (2)
N2	0.0178 (3)	0.0154 (3)	0.0136 (3)	−0.0040 (2)	−0.0021 (2)	−0.0016 (2)
C1	0.0178 (3)	0.0150 (3)	0.0164 (3)	−0.0050 (3)	−0.0036 (2)	0.0015 (2)
C2	0.0202 (4)	0.0188 (4)	0.0247 (4)	−0.0013 (3)	−0.0048 (3)	0.0011 (3)
C3	0.0249 (4)	0.0237 (4)	0.0270 (4)	−0.0130 (3)	−0.0048 (3)	0.0047 (3)
C4	0.0170 (3)	0.0150 (3)	0.0168 (3)	−0.0064 (3)	−0.0035 (2)	−0.0003 (2)
C5	0.0210 (4)	0.0187 (3)	0.0206 (3)	−0.0051 (3)	−0.0014 (3)	−0.0046 (3)
C6	0.0208 (4)	0.0244 (4)	0.0233 (4)	−0.0120 (3)	−0.0062 (3)	0.0016 (3)
C7	0.0162 (3)	0.0157 (3)	0.0161 (3)	−0.0040 (3)	−0.0025 (2)	−0.0007 (2)
C8	0.0245 (4)	0.0174 (3)	0.0230 (4)	−0.0074 (3)	−0.0045 (3)	0.0015 (3)
C9	0.0173 (3)	0.0250 (4)	0.0210 (3)	−0.0063 (3)	−0.0034 (3)	−0.0012 (3)
C10	0.0204 (3)	0.0195 (3)	0.0137 (3)	−0.0065 (3)	−0.0022 (2)	−0.0017 (2)
C11	0.0224 (4)	0.0285 (4)	0.0217 (4)	−0.0083 (3)	0.0026 (3)	−0.0047 (3)
C12	0.0296 (4)	0.0261 (4)	0.0206 (4)	−0.0125 (3)	−0.0046 (3)	−0.0049 (3)
B1	0.0179 (4)	0.0151 (4)	0.0166 (3)	−0.0048 (3)	−0.0022 (3)	−0.0015 (3)

### Bis(diisopropylamino)fluoroborane Geometric parameters (Å, º)

**Table:**

F1—B1	1.3650 (9)	C6—H6A	0.9800
N1—B1	1.4227 (10)	C6—H6B	0.9800
N1—C4	1.4676 (9)	C6—H6C	0.9800
N1—C1	1.4779 (9)	C7—C8	1.5310 (11)
N2—B1	1.4206 (10)	C7—C9	1.5343 (10)
N2—C7	1.4701 (9)	C7—H7	1.0000
N2—C10	1.4812 (9)	C8—H8A	0.9800
C1—C3	1.5272 (11)	C8—H8B	0.9800
C1—C2	1.5280 (11)	C8—H8C	0.9800
C1—H1	1.0000	C9—H9A	0.9800
C2—H2A	0.9800	C9—H9B	0.9800
C2—H2B	0.9800	C9—H9C	0.9800
C2—H2C	0.9800	C10—C11	1.5266 (11)
C3—H3A	0.9800	C10—C12	1.5298 (11)
C3—H3B	0.9800	C10—H10	1.0000
C3—H3C	0.9800	C11—H11A	0.9800
C4—C5	1.5284 (11)	C11—H11B	0.9800
C4—C6	1.5326 (10)	C11—H11C	0.9800
C4—H4	1.0000	C12—H12A	0.9800
C5—H5A	0.9800	C12—H12B	0.9800
C5—H5B	0.9800	C12—H12C	0.9800
C5—H5C	0.9800		
			
B1—N1—C4	123.84 (6)	H6B—C6—H6C	109.5
B1—N1—C1	121.01 (6)	N2—C7—C8	113.11 (6)
C4—N1—C1	115.11 (6)	N2—C7—C9	112.27 (6)
B1—N2—C7	123.64 (6)	C8—C7—C9	110.19 (6)
B1—N2—C10	120.53 (6)	N2—C7—H7	107.0
C7—N2—C10	115.66 (6)	C8—C7—H7	107.0
N1—C1—C3	111.48 (6)	C9—C7—H7	107.0
N1—C1—C2	113.89 (6)	C7—C8—H8A	109.5
C3—C1—C2	111.49 (7)	C7—C8—H8B	109.5
N1—C1—H1	106.5	H8A—C8—H8B	109.5
C3—C1—H1	106.5	C7—C8—H8C	109.5
C2—C1—H1	106.5	H8A—C8—H8C	109.5
C1—C2—H2A	109.5	H8B—C8—H8C	109.5
C1—C2—H2B	109.5	C7—C9—H9A	109.5
H2A—C2—H2B	109.5	C7—C9—H9B	109.5
C1—C2—H2C	109.5	H9A—C9—H9B	109.5
H2A—C2—H2C	109.5	C7—C9—H9C	109.5
H2B—C2—H2C	109.5	H9A—C9—H9C	109.5
C1—C3—H3A	109.5	H9B—C9—H9C	109.5
C1—C3—H3B	109.5	N2—C10—C11	111.40 (6)
H3A—C3—H3B	109.5	N2—C10—C12	113.43 (6)
C1—C3—H3C	109.5	C11—C10—C12	111.42 (7)
H3A—C3—H3C	109.5	N2—C10—H10	106.7
H3B—C3—H3C	109.5	C11—C10—H10	106.7
N1—C4—C5	112.25 (6)	C12—C10—H10	106.7
N1—C4—C6	112.37 (6)	C10—C11—H11A	109.5
C5—C4—C6	110.44 (6)	C10—C11—H11B	109.5
N1—C4—H4	107.2	H11A—C11—H11B	109.5
C5—C4—H4	107.2	C10—C11—H11C	109.5
C6—C4—H4	107.2	H11A—C11—H11C	109.5
C4—C5—H5A	109.5	H11B—C11—H11C	109.5
C4—C5—H5B	109.5	C10—C12—H12A	109.5
H5A—C5—H5B	109.5	C10—C12—H12B	109.5
C4—C5—H5C	109.5	H12A—C12—H12B	109.5
H5A—C5—H5C	109.5	C10—C12—H12C	109.5
H5B—C5—H5C	109.5	H12A—C12—H12C	109.5
C4—C6—H6A	109.5	H12B—C12—H12C	109.5
C4—C6—H6B	109.5	F1—B1—N2	116.05 (7)
H6A—C6—H6B	109.5	F1—B1—N1	115.03 (6)
C4—C6—H6C	109.5	N2—B1—N1	128.91 (7)
H6A—C6—H6C	109.5		
			
B1—N1—C1—C3	−58.17 (9)	B1—N2—C10—C11	−57.08 (9)
C4—N1—C1—C3	124.16 (7)	C7—N2—C10—C11	127.52 (7)
B1—N1—C1—C2	69.05 (9)	B1—N2—C10—C12	69.60 (9)
C4—N1—C1—C2	−108.62 (7)	C7—N2—C10—C12	−105.80 (8)
B1—N1—C4—C5	117.02 (8)	C7—N2—B1—F1	154.87 (7)
C1—N1—C4—C5	−65.38 (8)	C10—N2—B1—F1	−20.15 (10)
B1—N1—C4—C6	−117.78 (8)	C7—N2—B1—N1	−25.31 (12)
C1—N1—C4—C6	59.82 (8)	C10—N2—B1—N1	159.67 (8)
B1—N2—C7—C8	117.08 (8)	C4—N1—B1—F1	156.92 (7)
C10—N2—C7—C8	−67.68 (8)	C1—N1—B1—F1	−20.55 (10)
B1—N2—C7—C9	−117.45 (8)	C4—N1—B1—N2	−22.91 (12)
C10—N2—C7—C9	57.79 (8)	C1—N1—B1—N2	159.63 (7)
